# First record of mitochondrial genome of *Scincella reevesii* (Squamata: Scincidae) and phylogenetic analysis

**DOI:** 10.1080/23802359.2021.1875903

**Published:** 2021-02-15

**Authors:** Jun Zhong, Kun Guo, Li Ma

**Affiliations:** a College of Life Sciences, Jiangsu Key Laboratory for Biodiversity and Biotechnology, Nanjing Normal University, Nanjing, Jiangsu, China; b Faculty of Ecology, Lishui University, Lishui, Zhejiang, China

**Keywords:** Reptilia Squamata, *Scincella reevesii*, mitogenome phylogenetic analysis

## Abstract

We sequenced and annotated the nearly complete mitochondrial genome (mitogenome) of *Scincella reevesii* (Squamata: Scincidae). This mitogenome was 14,106 bp in size and encoded 13 protein-coding genes, two ribosomal RNA genes, and 22 transfer RNA genes. The most common start codon is ATG, the most common termination codon is TAA and five genes have incomplete termination codon T. The overall nucleotide composition was 32.0% of A, 14.3% of G, 26.1% of T, and 27.6% of C. The data will increase the basic information of Scincidae phylogenetic research and can help to better understand the phylogenetic status of *S. reevesii* in Squamata.

*Scincella reevesii* (Gray, 1838) (Squamata: Scincidae) is a small reptile, which is widely distributed in China. In the present study, we sequenced the mitochondrial genome of *S. reevesii* (GenBank Accession No. MN832615), which will help to better understand the phylogenetic status of this species in Squamata.

We collected *S. reevesii* samples in Zhaoqing, China (112°56′N, 23°09′E). The collected specimens were stored in 95% ethanol at temperature −20 °C and deposited in the Research Center of Herpetology, Nanjing Normal University, Nanjing, the Accession Number of the specimen was NB2017030715. Whole genomic DNA was extracted from tail tissues using a Wizard^®^ Genomic DNA Purification Kit (Promega, Madison, WI) according to the manufacturer’s instructions. The genomic DNA was sequenced using the Hiseq2000 platform (Illumina Inc., San Diego, CA). The mitogenome of *Scincella huanrenensis* (GenBank Accession No. KU507306) was employed as the reference sequence (Park et al. 2016). Mitochondrial genome was assembled by Geneious 9.0.4 (https://www.geneious.com), and annotated using MITOS Web Server (Bernt et al. 2013).

We obtained partial mitogenome of *S. reevesii* with 15,424 bp long. The region that we failed to sequence was between *trnP* and *trnF*, and generally contained a putative full-length control region. This mitogenome encoded 13 protein-coding genes (PCGs), 22 tRNAs, and two ribosomal RNA unit genes (*rrnL* and *rrnS*). The overall nucleotide composition was 32.0% of A, 14.3% of G, 26.1% of T, and 27.6% of C. Twelve PCGs started with typical ATG codon, whereas the *cox1* gene appeared to start with GTG. Eight PCGs (*ND1*, *COI*, *ATP8*, *ATP6*, *ND4L*, *ND5*, *ND6*, and *Cytb*) end with complete stop codons (TAA, AGA, and AGG), and the other five genes end with T as the incomplete stop codons, which were presumably completed as TAA by post transcriptional polyadenylation (Anderson et al. 1981).

To validate the phylogenetic position of *S. reevesii* in Scincidae family, a total of 12 PCGs from mitogenomes of Scincidae were obtained from GenBank, *Takydromus sexlineatus* and *Takydromus wolteri* in the genus of *Takydromus* of Lacertidae family were used as out-group. The maximum-likelihood (ML) tree was constructed using MEGA7.0 (Kumar et al. 2016) and inferred by bootstrap-ping with 1000 replicates. As shown in Figure 1, phylogenetic analysis showed *S. reevesii* was positioned in the genus of *Scincella* of Scincidae family, and had a closer relationship with three species within the genus *Scincella*, including *Scincella huanrenensis*, *Scincella modesta*, and *Scincella vandenburghi*.

**Figure 1.**
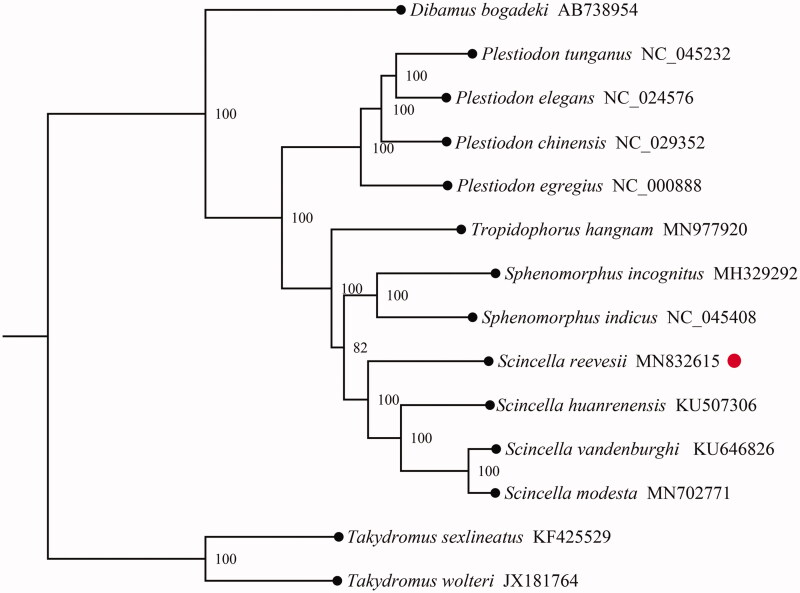
Phylogenetic tree obtained from maximum-likelihood (ML) analysis based on 14 concatenated mitochondrial PCGs. Numbers on node are posterior probability (PP).

## Data Availability

The genome sequence data that support the findings of this study are openly available in GenBank of NCBI at https://www.ncbi.nlm.nih.gov/ under the accession no. MN832615. The associated sequencing project is available in SRA and Bio-Sample, reference no. SUB8608459 and PRJNA680199.
